# Psychometric Properties of the Concise Associated Symptom Tracking Scale and Validation of Clinical Utility in the EMBARC Study

**DOI:** 10.1176/appi.prcp.20190041

**Published:** 2020-09-09

**Authors:** Abu Minhajuddin, Manish K. Jha, Cherise Chin Fatt, Madhukar H. Trivedi

**Affiliations:** ^1^ Center for Depression Research and Clinical Care University of Texas Southwestern Medical Center Dallas; ^2^ Department of Psychiatry Icahn School of Medicine at Mount Sinai New York

**Keywords:** irritability, major depressive disorder, anxiety, antidepressant treatment, outcome prediction, prognostic tool

## Abstract

**Objective:**

The authors aimed to evaluate psychometric properties of the Concise Associated Symptom Tracking (CAST) Scale and validate the clinical utility of measuring irritability by updating and replicating a previously published outcome calculator from the Combining Medications to Enhance Depression Outcomes (CO‐MED) trial.

**Methods:**

Participants were 292 adults from the Establishing Moderators and Biosignatures of Antidepressant Response in Clinical Care (EMBARC) study who had completed the CAST scale at baseline. The scale's five‐domain (irritability, anxiety, mania, insomnia, and panic) structure was evaluated with confirmatory factor analysis. Correlations with other clinical measures were used to confirm convergent and divergent validity. Logistic regression analyses from CO‐MED were used to estimate individual outcomes in EMBARC.

**Results:**

Cronbach's alpha for the CAST scale was 0.78. Model fit for the five‐domain structure was adequate (goodness of fit index=0.93, comparative fit index=0.92, root mean square error of approximation=0.06). Scores on irritability, anxiety, panic, insomnia, and mania were correlated with scores on the Anger Attack Questionnaire irritability item (r_s_=0.50), Hamilton Rating Scale for Depression anxiety subscale (r_s_=0.24), Mood and Anxiety Symptoms Questionnaire anxious arousal scale (r_s_=0.44), Quick Inventory of Depressive Symptomatology Self‐Report insomnia items (r_s_=0.38), and Altman Self‐Rating Mania Scale (r_s_=0.39), respectively. Individual outcomes of remission (area under the curve [AUC]=0.805) and no meaningful benefit (AUC=0.779) were predicted with high accuracy among EMBARC participants using their baseline and week 4 scores for depression and irritability and model estimates from CO‐MED.

**Conclusions:**

Measuring irritability may help predict clinical course. The CAST scale is a valid measure of depression‐associated symptoms, including irritability.

## HIGHLIGHTS

- Symptoms of anxiety, irritability, panic, and mania are often associated with depression.
- The Concise Associated Symptom Tracking Scale is a valid measure of these depression‐associated symptoms.
- Measuring irritability along with overall depression severity may help predict clinical course.

Patients diagnosed as having major depressive disorder experience a range of symptoms and functional impairments (1, 2, 3, 4, 5). Irritability in particular remains understudied among adults with the disorder (6, 7). Although irritability is reported by more than half of adult patients with major depression (6, 7), it is neither included as a diagnostic criterion in the *DSM‐5* (8) nor assessed by commonly used measures of depression severity (9, 10). Recently, the Concise Associated Symptom Tracking (CAST) Scale (11) was used to demonstrate the clinical utility of measuring irritability (12). That research found that irritability improved early (from baseline to week 4) with antidepressant treatment and that early improvement predicted higher rates of remission (no or minimal depression) and lower rates of no meaningful benefit (<30% reduction in depression) at week 8, independent of changes in depression severity (12). Finally, that research used baseline and week 4 measures of irritability and depression to develop an interactive calculator in one sample (Combining Medications to Enhance Depression Outcomes [CO‐MED]) and to validate it in a separate sample of outpatients with major depression (Suicide Assessment and Methodology Study [SAMS]) (12).

In the present study, we sought to extend these previous findings by evaluating the CAST scale's psychometric properties and by validating its clinical utility in an unrelated sample of outpatients with major depression from the Establishing Moderators and Biosignatures of Antidepressant Response for Clinical Care for Depression (EMBARC) study. To test the CAST scale's psychometric properties, we evaluated its five‐domain (anxiety, irritability, mania, panic, and insomnia) structure with confirmatory factory analysis, measuring internal consistency with Cronbach's alpha coefficient, and demonstrating construct validity through correlation of the CAST domains with other clinical assessments at baseline. We then validated the CAST scale's clinical utility in measuring irritability as a symptom of major depression by updating the previously published CO‐MED calculator (12) and testing the accuracy of calculator in predicting individual‐level outcomes in a separate sample (EMBARC) of adult outpatients with major depression.

## METHODS

### Study Design and Participants

#### EMBARC study

As previously described (13, 14), the EMBARC study (NCT01407094) enrolled 309 participants with major depressive disorder at four sites. Of these participants, 10 were excluded because they were part of a feasibility sample, and three were randomly assigned but were then found ineligible for the study (13). Of the 296 participants randomly assigned to receive either sertraline or placebo, four did not complete the CAST scale at baseline. Thus, the modified intent‐to‐treat sample for the present study consisted of 292 participants with major depressive disorder. Institutional review boards at each site approved the EMBARC study, and all participants provided written informed consent prior to beginning any study related procedures. Inclusion and exclusion criteria for the EMBARC study have been described (13) in detail (https://clinicaltrials.gov/ct2/show/NCT01407094). Briefly, EMBARC participants were ages 18–65, met criteria for current episode of major depressive disorder on the Structured Clinical Interview for DSM‐IV Axis I Disorders, scored ≥14 on the 16‐item Quick Inventory of Depressive Symptomatology Self‐Report (QIDS‐SR) at both screening and randomization visits, did not meet criteria for a failed antidepressant trial during the current episode as measured by the Massachusetts General Hospital Antidepressant Treatment Response Questionnaire (15), and agreed to and were eligible for all biomarker procedures (electroencephalography, psychological testing, magnetic resonance imaging, and blood draws). Participants were excluded if they did not tolerate sertraline or bupropion in the past; were pregnant, breastfeeding, or planning to become pregnant; were medically or psychiatrically unstable; had ever met criteria for psychotic and/or bipolar disorder; had experienced substance abuse in the past 2 months or substance dependence in past the 6 months; or were taking prohibited concomitant medications (antipsychotic, anticonvulsant, mood stabilizers, central nervous system stimulants, daily use of benzodiazepines or hypnotics, or antidepressants).

#### CO‐MED trial

For the present study, we used data from the CO‐MED trial participants to update the previously published logistic regression analyses of remission and no meaningful benefit as clinical outcomes (12). The CO‐MED trial (16) recruited from six primary and nine psychiatric sites 18–75‐year‐old treatment‐seeking outpatients with major depressive disorder (N=665) and at least moderately severe (score of ≥16 on the 17‐item Hamilton Depression Rating Scale [HAMD‐17]) nonpsychotic chronic or recurrent depression. All participants provided written informed consent, and institutional review board approval was granted from each participating site (16). Detailed eligibility criteria have been reported (16) and are available on the Internet (https://clinicaltrials.gov/ct2/show/NCT00590863). At baseline, participants were randomly assigned to treatment with either escitalopram plus placebo, sustained‐release bupropion plus escitalopram, or extended‐release venlafaxine plus mirtazapine. Postrandomization visits were conducted at weeks 1, 2, 4, 6, 8, 10, and 12 for the acute phase and at weeks 16, 20, 24, and 28 for the continuation phase.

### Measurements

#### HAMD‐17

Clinicians conducted the structured interview (17) for HAMD‐17 to assess depression severity of patients at each visit of the EMBARC study. Previous reports have found concurrent validity between the HAMD‐17 and other measures of depression severity, such as the 30‐item Inventory of Depressive Symptomatology–Clinician Rated (IDS‐C) (18). Six items of the HAMD‐17 (psychic anxiety, somatic anxiety, gastrointestinal somatic symptoms, general somatic symptoms, hypochondriasis, and insight) (19, 20) have been used to establish an anxiety subscale.

#### Quick Inventory of Depressive Symptomatology Self–Report (QIDS‐SR)

The 16 items (each scored from 0 to 3) of the QIDS‐SR are based on the nine symptom domains of major depressive disorder (10). Total scores for this tool range from 0 to 27. The QIDS‐SR correlates highly with the HAMD‐17 (r=0.86–0.93) and has high inter‐item correlation (Cronbach's α=0.86–0.87) (10). Because the first three items of the QIDS‐SR assess insomnia, we combined them to assess severity of insomnia for the present study. Participants completed the QIDS‐SR only during screening and at the baseline visit of EMBARC.

#### Concise Associated Symptom Tracking Scale Self‐Report

The 16 items of the CAST Self‐Report Scale assesses symptoms across five domains: anxiety (three items, range 3–15), irritability (five items, range 5–25), mania (four items, range 4–20), insomnia (two items, range 2–10), and panic (two items, range 2–10). Each individual item is rated on a 5‐point Likert scale as 1, strongly disagree; 2, disagree; 3, neither agree nor disagree; 4, agree; or 5, strongly agree (11). In previous reports, Cronbach's alpha for the CAST scale was 0.78 (11) and was 0.83, 0.87, 0.84, 0.92, and 0.92 for the irritability, anxiety, mania, insomnia, and panic domains, respectively (21).

#### Altman Self‐Rating Mania Scale (ASRM)

The ASRM is a five‐item self‐reported scale designed to evaluate for the presence and severity of manic and hypomanic symptoms over the past 7 days. Each item consists of five possible responses, with scores ranging from 0 to 4. Item scores are added for a total score; 0 is the lowest possible score and 20 is the maximum possible (22).

#### Mood and Anxiety Symptoms Questionnaire (MASQ)

The 30‐item short‐form adaptation of the MASQ (23) was used to assess the participants' negative affect, positive affect, and somatic arousal. Each item of the MASQ covers a recall period of 1 week and is rated on a 5‐point Likert scale from 1, not at all, to 5, extremely. Cronbach's alpha for the 30‐item MASQ in a previous study ranged from 0.85 to 0.95 (23). Furthermore, factor analyses have confirmed the 30‐item MASQ's three‐factor structure with the following three scales: general distress, anhedonic depression, and anxious arousal (23).

#### Anger attacks question

At the baseline visit of the EMBARC study, participants completed the Massachusetts General Hospital Anger Attack Questionnaire (AAQ) (24). We used the responses to the first item of the AAQ, “Over the past six months, have you felt irritable or easily angered,” to test convergent validity of the irritability domain.

### Adaptation of the CO‐MED Outcome Calculator

In the CO‐MED trial, separate logistic regression analyses were used to predict individual outcomes of remission (no or minimal depression) and no meaningful benefit (<30% reduction from baseline) at week 8 by using scores for depression (QIDS‐C) and irritability (CAST‐IRR [i.e., the CAST scale's irritability domain]) at baseline and week 4 (12). Model β estimates from these logistic regression analyses were then used in a separate sample (the SAMS study) of outpatients with depression to estimate individual‐level probability of remission or no meaningful benefit to build an interactive calculator (12). For the present report, we had to update the logistic regression analyses used in the CO‐MED trial, because measures of depression severity differed between EMBARC (HAMD‐17) (13) and CO‐MED (IDS‐C and QIDS‐SR). We used a formula by Vittengl et al. (18) to convert IDS‐C scores to HAMD‐17 scores: HAMD‐17=0.11+0.53×(IDS‐C). (The results of our logistic regression analyses, which used converted HAMD‐17 scores as the measure of depression severity and CAST‐IRR as the measure of irritability, are available in the online supplement to this article.)

### Statistical Analysis

As previously mentioned, the modified intent‐to‐treat sample for the present study consisted of all EMBARC study participants who were randomly assigned to receive sertraline or placebo and who had completed the CAST scale at baseline of EMBARC (N=292). Psychometric properties of the CAST scale were validated with EMBARC participants only. We used a confirmatory factor analysis implemented in PROC CALIS in SAS to validate the scale's five‐domain structure. We defined acceptable model fit a priori as a goodness‐of‐fit index ≥0.90, comparative fit index ≥0.90, and root mean square error of approximation ≤0.08 (25). We estimated the Pearson product‐moment correlation coefficient (r) to evaluate association among the scale's five domains. We used separate item response theory (IRT) analyses, based on a graded response model (26) for each domain, to evaluate the performance of individual items. The slope for each item provides an estimate of that item's ability to discriminate between differences in levels of specific domains, and the thresholds indicate the item's sensitivity at difference levels. Specifically, for each item on the scale, threshold 1 compared selecting 1 versus 2, 3, 4, or 5; threshold 2 compared selecting 1 or 2 versus 3, 4, or 5; threshold 3 compared selecting 1, 2, or 3 versus 4 or 5; and threshold 4 compared selecting 1, 2, 3, or 4 versus 5 (27, 28). Cronbach's alpha coefficient was calculated to evaluate the internal consistency of the 16‐item CAST scale and the individual domains (29). We also calculated the Spearman rank‐order correlation coefficient (r_s_) between the five domains and our clinical assessments for convergent and divergent validity.

To validate the scale's clinical utility, we updated previously described logistic regression analyses from the CO‐MED trial, which had remission and no meaningful benefit at week 8 as outcomes (12), by replacing QIDS‐C with computed HAMD‐17, using the formula HAMD‐17=0.11+0.53×(IDS‐C) per Vittengl et al. (18). For EMBARC study participants with HAMD‐17 scores available at week 8 (N=240), remission and no meaningful benefit at week 8 were defined as HAMD‐17 ≤7 and <30% reduction in HAMD‐17 from baseline, respectively. By using the intercept and β estimates from updated logistic regression analyses of the CO‐MED trial and baseline and baseline‐to‐week 4 percentage changes in CAST‐IRR and HAMD‐17 scores from the EMBARC study, we estimated individual probabilities of remission and no meaningful benefit for the present study. We then calculated individual level probability (p) of remission and no meaningful benefit among our EMBARC participants by multiplying the β estimates obtained from the CO‐MED trial with the observed scores in the EMBARC study to solve the following equation: log(p/1–p)=intercept+β_baseline depression from CO‐MED_×(baseline depression in EMBARC)+β_baseline irritability from CO‐MED_×(baseline irritability in EMBARC)+β _percent change in depression from CO‐MED_×(percent change in depression in EMBARC)+β_percent change in irritability from CO‐MED_×(percent change in irritability in EMBARC). We then compared these estimated probabilities with observed occurrence of these outcomes by using receiver operating characteristic (ROC) curves. We conducted all analyses with SAS, version 9.4; threshold of significance was set at p<0.05.

## RESULTS

At baseline, mean±SD scores for the CAST scale's five domains (irritability, anxiety, mania, insomnia, and panic) were 16.32±3.96, 8.84±2.66, 7.57±2.65, 6.64±2.06, and 4.54±2.00, respectively. Detailed frequency of responses to individual items of the scale is shown in Table 1 along with the mean for these items. Furthermore, at week 8 of the EMBARC study, 35.8% of participants (N=86 of 240) attained remission (N=45 of 114 receiving sertraline; N=41 of 126 receiving placebo) and 40.8% (N=98 of 240) experienced no meaningful benefit (N=40 of 114 receiving sertraline; N=58 of 126 receiving placebo).

**TABLE 1:** Responses by EMBARC participants (N=292) to individual items of the Concise Associated Symptom Tracking (CAST) Scale at baseline^a^

Item^b^	CAST domain	Strongly disagree		Disagree		Neither agree nor disagree		Agree		Strongly agree			
		N	%	N	%	N	%	N	%	N	%	M	SD
1. I feel anxious all the time	Anxiety	22	8	49	17	62	21	120	41	39	13	3.36	1.14
2. I have been feeling really good lately	Mania	117	40	120	41	40	14	13	4	2	1	1.83	.86
3. I feel as if I am going to have a heart attack	Panic	131	45	79	27	43	15	37	13	2	1	1.97	1.08
4. I wish people would just leave me alone	Irritability	23	8	61	21	86	29	92	32	30	10	3.16	1.11
5. I have been having more trouble sleeping than usual	Insomnia	18	6	54	19	56	19	111	38	53	18	3.43	1.16
6. I am feeling restless, as if I have to move constantly	Anxiety	39	13	80	27	66	23	88	30	19	7	2.89	1.17
7. I suddenly feel very confident	Mania	153	52	108	37	19	7	9	3	3	1	1.63	.81
8. I am more talkative than normal	Mania	119	41	124	43	26	9	18	6	5	2	1.85	.94
9. I feel very uptight	Irritability	28	10	51	18	57	20	122	42	33	11	3.28	1.17
10. I find myself saying or doing things without thinking	Irritability	30	10	101	35	65	22	83	28	13	4	2.82	1.09
12. I can feel my heart racing	Panic	62	21	96	33	50	17	75	26	9	3	2.56	1.17
13. Lately everything seems to be annoying me	Irritability	12	4	44	15	611	21	130	45	45	15	3.52	1.05
14. I slept very little last night	Insomnia	28	10	80	27	38	13	95	33	51	18	3.21	1.28
15. I cannot sit still	Anxiety	47	16	109	37	68	23	54	19	14	5	2.59	1.11
16. I find people get on my nerves easily	Irritability	7	2	57	20	50	17	121	41	57	20	3.56	1.09
17. I have been having lots of great ideas	Mania	78	27	103	35	78	27	29	10	4	1	2.24	1.00

^a^ EMBARC, Establishing Moderators and Biosignatures of Antidepressant Response in Clinical Care study.

^b^ Item numbers refer to numbers originally reported for the 17‐item CAST Scale (11). The 16‐item CAST Scale used in the EMBARC study had excluded item 11 from the original 17‐item scale because it had loaded on two factors.

### Validation of the CAST Scale's Psychometric Properties

#### Five‐domain structure

In confirmatory factor analyses, goodness of fit, comparative fit index, and root mean square error of approximation were 0.93, 0.92, 0.06, respectively, for the CAST scale's five‐domain structure. Because three out of three a priori defined criteria were met, the model fit was deemed acceptable. The standardized factor loadings for the anxiety, irritability, mania, insomnia, and panic domains ranged from 0.36 to 0.85, 0.48 to 0.85, 0.45 to 0.78, 0.62 to 0.68, and 0.61 to 0.93, respectively (Table 2). The anxiety domain was moderately correlated with the other domains (r=0.30–0.43). The irritability domain was associated only with anxiety (r=0.43) and panic (r=0.34). (See Table 2 for correlations among the five domains.)

**TABLE 2:** Standardized factor loadings and Pearson's correlation coefficients for the Concise Associated Symptom Tracking Scale's five‐domain structure^a^

Item/domain	Anxiety	Irritability	Mania	Insomnia	Panic
**Standardized factor loadings**					
I feel anxious all the time	.36				
I have been feeling really good lately			.45		
I feel as if I am going to have a heart attack					.61
I wish people would just leave me alone		.48			
I have been having more trouble sleeping than usual				.62	
I am feeling restless, as if I have to move constantly	.85				
I suddenly feel very confident			.78		
I am more talkative than normal			.70		
I feel very uptight		.54			
I find myself saying or doing things without thinking		.54			
I can feel my heart racing					.93
Lately everything seems to be annoying me		.79			
I slept very little last night				.68	
I cannot sit still	.79				
I find people get on my nerves easily		.85			
I have been having lots of great ideas			.51		
**Pearson's correlation coefficient**					
Anxiety	.1				
Irritability	.43	.1			
Mania	.39	.13	.1		
Insomnia	.30	.08	.07	.1	
Panic	.30	.45	.22	.34	.1

^a^ Factor loading of individual items and correlation among individual domains were obtained from confirmatory factor analysis.

#### IRT analyses

In polychoric correlation matrices from IRT analyses, only the first factor of each domain had an eigenvalue exceeding 1.00, supporting the unidimensionality of each domain. The eigenvalues of the first factors of the anxiety, irritability, mania, panic, and insomnia domains were 1.95, 2.86, 2.47, 1.66, and 1.50, respectively. Furthermore, for each domain, the slope of all items exceeded 1.0 (excluding the first item of the anxiety domain), indicating that these items provided adequate discrimination. Table 3 presents the item slopes and thresholds of difficulty for each domain.

**TABLE 3:** Slopes and thresholds of difficulty of items on the CAST Scale^a^

		Threshold			
**Item**	**Slope**	1	2	3	4
Anxiety (3 items)					
1. I feel anxious all the time	.73	−3.75	−1.72	−.26	2.83
6. I am feeling restless, as if I have to move constantly	3.50	−1.24	−.27	.38	1.71
15. I cannot sit still	2.73	−1.18	.10	.87	2.00
Irritability (5 items)					
4. I wish people would just leave me alone	1.19	−2.62	−1.02	.32	2.32
9. I feel very uptight	1.23	−2.26	−1.03	−.14	2.10
10. I find myself saying or doing things without thinking	1.22	−2.19	−.25	.71	3.01
13. Lately everything seems to be annoying me	2.76	−2.08	−1.01	−.30	1.18
16. I find people get on my nerves easily	4.49	−2.17	−.81	−.29	.90
Mania (4 items)					
2. I have been feeling really good lately	1.26	−.40	1.47	2.78	4.51
7. I suddenly feel very confident	3.18	.11	1.40	1.95	2.61
8. I am more talkative than normal	2.30	−.25	1.22	1.76	2.64
17. I have been having lots of great ideas	1.34	−.97	.55	1.96	3.75
Panic (2 items)					
3. I feel as if I am going to have a heart attack	5.92	−.14	.57	1.15	2.70
12. I can feel my heart racing	1.71	−1.11	.15	.78	2.77
Insomnia (2 items)					
5. I have been having more trouble sleeping than usual	1.58	−2.30	−1.02	−.26	1.33
14. I slept very little last night	2.16	−1.66	−.44	−.02	1.20

^a^ The parameters shown for each threshold were obtained from separate item response theory analyses for each domain. Item numbers refer to numbers originally reported for the 17‐item Concise Associated Symptom Tracking (CAST) Scale (11).

#### Internal consistency

Cronbach's alpha for the 16‐item CAST scale was 0.78, whereas those for the irritability, anxiety, mania, insomnia, and panic domains were 0.77, 0.68, 0.71, 0.59, and 0.72, respectively.

#### Construct validity

The irritability, anxiety, insomnia, and panic domains were positively, albeit modestly, correlated with measures of overall depression severity, namely the QIDS‐SR (r_s_=0.17–0.24) and HAMD‐17 (r_s_=0.11–0.34). The mania domain was not significantly correlated with these measures of depression severity (Table 4). The anxiety domain was positively correlated with scores on the HAMD‐17 anxiety subscale (r_s_=0.24) and the MASQ anxious arousal scale (r_s_=0.30). Items related to irritability on other scales, such as the AAQ and MASQ, were significantly correlated only with the CAST scale's irritability domain (r_s_=0.50 and r_s_=0.45, respectively). The irritability domain was poorly correlated with scales for mania (r_s_=0.06 with ASRM) and insomnia (r_s_=0.09 with the sum of the insomnia items on the QIDS‐SR). The mania, insomnia, and panic domains were positively correlated with the ASRM (r_s_=0.39), the sum of insomnia items on the QIDS‐SR (r_s_=0.38), and the MASQ's anxious arousal subscale (r_s_=0.44), respectively.

**TABLE 4:** **Spearman correlation coefficients of CAST Scale domains with baseline clinical characteristics of EMBARC study participants (N=292)** ^a^

Domain	Anxiety	Irritability	Mania	Insomnia	Panic
QIDS‐SR	.22	.24	−.06	.17	.22
17‐item Hamilton Rating Scale for Depression (HAMD‐17)	.18	.11	−.09	.34	.18
Irritability item of Anger Attack Questionnaire	.17	.50	.02	−.03	.12
Irritability item of MASQ	.17	.45	−.03	−.05	.10
Hamilton Rating Scale for Depression, anxiety scale	.24	.21	.05	.12	.22
Altman Self‐Rating Mania Scale	.14	.06	.39	.09	.06
QIDS‐SR, insomnia total	.09	.04	.05	.38	.12
MASQ, general distress scale	.20	.31	−.24	.04	.17
MASQ, anhedonic depression scale	−.02	.13	−.47	.08	.13
MASQ, anxious arousal scale	.30	.32	.08	.05	.44

^a^ CAST, Concise Associated Symptom Tracking scale; EMBARC, Establishing Moderators and Biosignatures of Antidepressant Response in Clinical Care study; QIDS‐SR, Quick Inventory of Depressive Symptomatology–Self‐Report version; MASQ, 30‐item Mood and Anxiety Symptom Questionnaire.

### Validation of Clinical Utility

**FIGURE 1:**
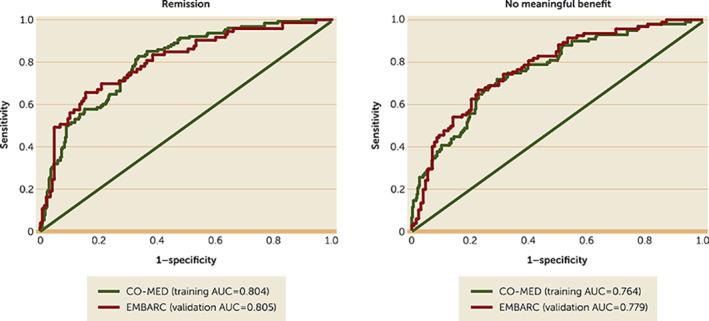
Receiver operating characteristic curves for remission and no meaningful benefit among EMBARC (N=221) and CO‐MED (N=431) participants^a^ ^a^Separate logistic regression analyses were conducted using data from the Combining Medications to Enhance Depression Outcomes (CO‐MED) trial with remission (17‐item Hamilton Depression Rating Scale [HAMD‐17] score ≤7) and no meaningful benefit (reduction in HAMD‐17 score <30% from baseline) at week 8 as outcomes and with baseline and baseline‐to‐week‐4 changes in HAMD‐17 and Concise Associated Symptom Tracking (CAST) Scale scores as independent variables. The β estimates for each independent variable in these logistic regression analyses from the CO‐MED trial (training sample) were multiplied by the observed scores in the Establishing Moderators and Biosignatures of Antidepressant Response in Clinical Care (EMBARC) study (validation sample) to solve the following equation: log(p/1‐p)=intercept+β_baseline depression from CO‐MED_×(baseline depression in EMBARC)+β_baseline irritability from CO‐MED_×(baseline irritability in EMBARC)+β_percent change in depression from CO‐MED_×(percent change in depression in EMBARC) + β_percent change in irritability from CO‐MED_×(percent change in irritability in EMBARC). The resultant estimated probabilities were then compared with the observed occurrence of these outcomes by using receiver operating characteristic curves. The areas under the curve (AUCs) were comparable for CO‐MED and EMBARC.

By using the baseline scores of the HAMD‐17 and CAST‐IRR along with the baseline‐to‐week‐4 changes in these measures among our EMBARC study participants and model estimates from the logistic regression models (see online supplement) in the CO‐MED trial (12), we found that individual‐level prediction of remission (area under the curve [AUC]=0.805) and no meaningful benefit (AUC=0.779) in the EMBARC study were similar to those of the CO‐MED trial (remission AUC=0.804; no meaningful benefit AUC=0.764) (Figure 1). This finding provides validation of the CO‐MED calculator in an independent sample.

## DISCUSSION

In this study of a large sample of adult outpatients with major depressive disorder, we found confirmatory evidence for the psychometric properties and five‐domain structure of the 16‐item CAST scale. Furthermore, we extended the clinical utility of CAST as a measure of irritability by updating and validating a previously reported individual‐level calculator for prediction of acute‐phase treatment outcomes of remission and no meaningful benefit.

Our findings are consistent with previous studies that have found the CAST scale to have sound psychometric properties (11, 21). Additionally, consistent with previous reports, we found moderate association between measures of irritability and anxiety (2, 30, 31). Future studies are needed to identify the shared versus unique components of these domains as well as the overlap between self‐reported symptoms of irritability and overt behavior, such as anger attacks (32). Strengths of this study include validity of the CAST scale's irritability domain as a self‐report measure of irritability and replication of its clinical utility by prediction of individual‐level acute‐phase treatment outcomes of remission and no meaningful benefit.

Although the multidimensional nature of anxiety among patients with major depressive disorder has been reported (2), similar studies of irritability and its related constructs of frustration and hostility are lacking among adult patients with major depression. Whereas presence of irritability at the index episode of major depression has been linked to greater subsequent functional impairments (7), future studies are needed to characterize the trajectories of treatment‐related changes in irritability with other outcomes, such as psychosocial functioning (33), work‐ and non‐work‐related productivity (1, 5, 34), and quality of life (35). Studies of irritability in adults with major depression must also consider the issue of bipolarity, because persistent irritability is one of the diagnostic criteria for mania and hypomania (8). Although the EMBARC and CO‐MED trials excluded patients with lifetime history of mania or hypomania as determined by structured diagnostic interview (13, 16), we have previously found high prevalence rates of subthreshold hypomanic symptoms among patients with major depression (3). However, we found no significant association between measures of mania (as measured by the CAST scale's mania domain as well as the ASRM) and irritability in this study or in a previous one (21). It is noteworthy that in developmental literature, irritability during childhood is associated with subsequent onset of unipolar depression and anxiety disorder but not of bipolar disorder (36, 37).

This study had several limitations. The findings were obtained through unplanned secondary analyses, and as such the analyses may not have been adequately powered. Validation of the psychometric properties of the CAST scale was not a primary goal of EMBARC. Hence, the EMBARC study design did not include separate measures of irritability, insomnia, and panic beyond the baseline visit. Furthermore, EMBARC's inclusion and exclusion criteria may make the findings less generalizable to all patients with major depressive disorder (38). When available, self‐report symptom rating measures (such as the CAST scale and the ASRM) were used instead of clinician‐rated versions, because self‐report measures can be implemented easily in large‐scale measurement‐based care approaches to manage depression in real‐world clinics (39, 40). Self‐report measures of depression severity were not available after the baseline visit in EMBARC (13). Whereas clinician and patient‐rated measures of depression severity have been shown to reflect the same symptom severity and change constructs (18), similar systematic assessments of clinician‐rated and self‐report versions of the CAST scale have not been done. Future studies should evaluate the clinical significance of differences in clinician‐rated versus self‐report measures of the CAST scale (41).

## CONCLUSIONS

In this large sample of adult outpatients with major depressive disorder, we found the 16‐item CAST scale to have good psychometric properties. Furthermore, we replicated findings that irritability is an important symptom domain, which can help predict individual‐level outcomes of remission or no meaningful benefit when combined with measures of depression severity. Thus, this study informs research on irritability among adults with major depressive disorder and guides clinical practice by improving the accuracy of clinical course prognostication.

## Supporting information

Supplementary Material

## References

[1] Jha MK , Minhajuddin A , Greer TL , et al: Early improvement in work productivity predicts future clinical course in depressed outpatients: findings from the CO‐MED trial. Am J Psychiatry 2016; 173:1196–1204 27523501 10.1176/appi.ajp.2016.16020176 PMC5895453

[2] Trombello JM , Pizzagalli DA , Weissman MM , et al: Characterizing anxiety subtypes and the relationship to behavioral phenotyping in major depression: results from the EMBARC study. J Psychiatr Res 2018; 102:207–215 29689518 10.1016/j.jpsychires.2018.04.003 PMC6097520

[3] Jha MK , Malchow AL , Grannemann BD , et al: Do baseline sub‐threshold hypomanic symptoms affect acute‐phase antidepressant outcome in outpatients with major depressive disorder? Preliminary findings from the randomized CO‐MED trial. Neuropsychopharmacology 2018; 43:2197–2203 30135556 10.1038/s41386-018-0180-z PMC6135801

[4] Bushnell DM , McCarrier KP , Bush EN , et al: Symptoms of major depressive disorder scale: performance of a novel patient‐reported symptom measure. Value Health 2019; 22:906–915 31426932 10.1016/j.jval.2019.02.010

[5] Jha MK , South C , Trivedi J , et al: Prediction of acute‐phase treatment outcomes by adding a single‐item measure of activity impairment to symptom measurement: development and validation of an interactive calculator from the STAR*D and CO‐MED trials. Int J Neuropsychopharmacol 2019; 22:339–348 30958879 10.1093/ijnp/pyz011 PMC6499251

[6] Fava M , Hwang I , Rush AJ , et al: The importance of irritability as a symptom of major depressive disorder: results from the National Comorbidity Survey Replication. Mol Psychiatry 2010; 15:856–867 19274052 10.1038/mp.2009.20 PMC3012558

[7] Judd LL , Schettler PJ , Coryell W , et al: Overt irritability/anger in unipolar major depressive episodes: past and current characteristics and implications for long‐term course. JAMA Psychiatry 2013; 70:1171–1180 24026579 10.1001/jamapsychiatry.2013.1957

[8] Diagnostic and Statistical Manual of Mental Disorders, 5th ed. Washington, DC, American Psychiatric Association, 2013

[9] Kroenke K , Spitzer RL , Williams JB : The PHQ‐9: validity of a brief depression severity measure. J Gen Intern Med 2001; 16:606–613 11556941 10.1046/j.1525-1497.2001.016009606.x PMC1495268

[10] Rush AJ , Trivedi MH , Ibrahim HM , et al: The 16‐Item Quick Inventory of Depressive Symptomatology (QIDS), Clinician Rating (QIDS‐C), and Self‐Report (QIDS‐SR): a psychometric evaluation in patients with chronic major depression. Biol Psychiatry 2003; 54:573–583 12946886 10.1016/s0006-3223(02)01866-8

[11] Trivedi MH , Wisniewski SR , Morris DW , et al: Concise Associated Symptoms Tracking Scale: a brief self‐report and clinician rating of symptoms associated with suicidality. J Clin Psychiatry 2011; 72:765–774 21733477 10.4088/JCP.11m06840

[12] Jha MK , Minhajuddin A , South C , et al: Irritability and its clinical utility in major depressive disorder: prediction of individual‐level acute‐phase outcomes using early changes in irritability and depression severity. Am J Psychiatry 2019; 176:358–366 30922100 10.1176/appi.ajp.2018.18030355

[13] Trivedi MH , McGrath PJ , Fava M , et al: Establishing moderators and biosignatures of antidepressant response in clinical care (EMBARC): rationale and design. J Psychiatr Res 2016; 78:11–23 27038550 10.1016/j.jpsychires.2016.03.001 PMC6100771

[14] Trivedi MH , South C , Jha MK , et al: A novel strategy to identify placebo responders: prediction index of clinical and biological markers in the EMBARC trial. Psychother Psychosom 2018; 87:285–295 30110685 10.1159/000491093 PMC9764260

[15] Chandler GM , Iosifescu DV , Pollack MH , et al: RESEARCH: validation of the Massachusetts General Hospital Antidepressant Treatment History Questionnaire (ATRQ). CNS Neurosci Ther 2010; 16:322–325 19769599 10.1111/j.1755-5949.2009.00102.x PMC6493891

[16] Rush AJ , Trivedi MH , Stewart JW , et al: Combining medications to enhance depression outcomes (CO‐MED): acute and long‐term outcomes of a single‐blind randomized study. Am J Psychiatry 2011; 168:689–701 21536692 10.1176/appi.ajp.2011.10111645

[17] Williams JB : A structured interview guide for the Hamilton Depression Rating Scale. Arch Gen Psychiatry 1988; 45:742–747 3395203 10.1001/archpsyc.1988.01800320058007

[18] Vittengl JR , Clark LA , Kraft D , et al: Multiple measures, methods, and moments: a factor‐analytic investigation of change in depressive symptoms during acute‐phase cognitive therapy for depression. Psychol Med 2005; 35:693–704 15918346 10.1017/s0033291704004143 PMC1410810

[19] Cleary P , Guy W : Factor analysis of the Hamilton Depression Scale. Drugs Exp Clin Res 1977; 1:115–120

[20] Fava M , Rush AJ , Alpert JE , et al: Difference in treatment outcome in outpatients with anxious versus nonanxious depression: a STAR*D report. Am J Psychiatry 2008; 165:342–351 18172020 10.1176/appi.ajp.2007.06111868

[21] Jha MK , Minhajuddin A , South C , et al: Worsening anxiety, irritability, insomnia, or panic predicts poorer antidepressant treatment outcomes: clinical utility and validation of the Concise Associated Symptom Tracking (CAST) Scale. Int J Neuropsychopharmacol 2018; 21:325–332 29182724 10.1093/ijnp/pyx097 PMC5888105

[22] Altman EG , Hedeker D , Peterson JL , et al: The Altman Self‐Rating Mania Scale. Biol Psychiatry 1997; 42:948–955 9359982 10.1016/S0006-3223(96)00548-3

[23] Wardenaar KJ , van Veen T , Giltay EJ , et al: Development and validation of a 30‐item short adaptation of the Mood and Anxiety Symptoms Questionnaire (MASQ). Psychiatry Res 2010; 179:101–106 20472297 10.1016/j.psychres.2009.03.005

[24] Fava M , Anderson K , Rosenbaum JF : “Anger attacks”: possible variants of panic and major depressive disorders. Am J Psychiatry 1990; 147:867–870 2356872 10.1176/ajp.147.7.867

[25] Hooper D , Coughlan J , Mullen M : Structural equation modelling: guidelines for determining model fit. The Electronic Journal of Business Research Methods 2008; 6:53–25

[26] Samejima F : Graded response model; in Handbook of Modern Item Response Theory. Edited by van der Linden, WJ , Hambleton, RK. New York, Springer, 1997

[27] Rush AJ , South CC , Jha MK , et al: Toward a very brief Quality of Life Enjoyment and Satisfaction Questionnaire. J Affect Disord 2019; 242:87–95 30173063 10.1016/j.jad.2018.08.052

[28] Rush AJ , Bernstein IH , Trivedi MH , et al: An evaluation of the Quick Inventory of Depressive Symptomatology and the Hamilton Rating Scale for Depression: a sequenced treatment alternatives to relieve depression trial report. Biol Psychiatry 2006; 59:493–501 16199008 10.1016/j.biopsych.2005.08.022 PMC2929841

[29] Cronbach LJ : Coefficient alpha and the internal structure of tests. Psychometrika. 1951; 16:297–334

[30] Cornacchio D , Crum KI , Coxe S , et al: Irritability and severity of anxious symptomatology among youth with anxiety disorders. J Am Acad Child Adolesc Psychiatry 2016; 55:54–61 26703910 10.1016/j.jaac.2015.10.007 PMC5340317

[31] Stoddard J , Tseng WL , Kim P , et al: Association of irritability and anxiety with the neural mechanisms of implicit face emotion processing in youths with psychopathology. JAMA Psychiatry 2017; 74:95–103 27902832 10.1001/jamapsychiatry.2016.3282 PMC6309540

[32] Pine DS : Heterogeneity in major depressive disorder: lessons from developmental research on irritability. Am J Psychiatry 2019; 176:331–332 31039634 10.1176/appi.ajp.2019.19020214

[33] Jha MK , Minhajuddin A , Greer TL , et al: Early improvement in psychosocial function predicts longer‐term symptomatic remission in depressed patients. PLoS One 2016; 11:e0167901 28030546 10.1371/journal.pone.0167901 PMC5193346

[34] Jha MK , Teer RB , Minhajuddin A , et al: Daily activity level improvement with antidepressant medications predicts long‐term clinical outcomes in outpatients with major depressive disorder. Neuropsychiatr Dis Treat 2017; 13:803–813 28352180 10.2147/NDT.S128407 PMC5359139

[35] Jha MK , Greer TL , Grannemann BD , et al: Early normalization of quality of life predicts later remission in depression: findings from the CO‐MED trial. J Affect Disord 2016; 206:17–22 27455354 10.1016/j.jad.2016.07.012

[36] Leibenluft E : Severe mood dysregulation, irritability, and the diagnostic boundaries of bipolar disorder in youths. Am J Psychiatry 2011; 168:129–142 21123313 10.1176/appi.ajp.2010.10050766 PMC3396206

[37] Riglin L , Eyre O , Thapar AK , et al: Identifying novel types of irritability using a developmental genetic approach. Am J Psychiatry 2019; 176:635–642 31256611 10.1176/appi.ajp.2019.18101134 PMC6677571

[38] Zimmerman M , Balling C , Chelminski I , et al: Have treatment studies of depression become even less generalizable? Applying the inclusion and exclusion criteria in placebo‐controlled antidepressant efficacy trials published over 20 years to a clinical sample. Psychother Psychosom 2019; 88:165–170 31096246 10.1159/000499917

[39] Jha MK , Grannemann BD , Trombello JM , et al: A structured approach to detecting and treating depression in primary care: VitalSign6 Project. Ann Fam Med 2019; 17:326–335 31285210 10.1370/afm.2418 PMC6827639

[40] Trivedi MH , Jha MK , Kahalnik F , et al: VitalSign6: a primary care first (PCP‐first) model for universal screening and measurement‐based care for depression. Pharmaceuticals (Basel) 2019; 12:71 10.3390/ph12020071 PMC6630588 31091770

[41] Tada M , Uchida H , Suzuki T , et al: Baseline difference between patients' and clinicians' rated illness severity scores and subsequent outcomes in major depressive disorder: analysis of the Sequenced Treatment Alternatives to Relieve Depression data. J Clin Psychopharmacol 2014; 34:297–302 24743720 10.1097/JCP.0000000000000112 PMC3992483

