# Supplementary material for: Psychometric Properties of the Concise Associated Symptom Tracking Scale and Validation of Clinical Utility in the EMBARC Study
Source: Psychiatr Res Clin Pract. 2020 Sep 9;2(1):10–8. doi: 10.1176/appi.prcp.20190041 (PMC9175787; doi:10.1176/appi.prcp.20190041)
Supplement: Supplementary file 1 — Supplementary Material [file RCP2-2-10-s001.pdf]

Supplementary table ST1. Results of logistic regression analyses using computed HAMD-17 (cHAMD-17) in CO-MED trial

	Remission at week-8				No-meaningful-benefit at week-8			
	Estimate	SE	χ^2	p	Estimate	SE	χ^2	p
Intercept	-0.2635	0.6695	0.1549	0.6939	-0.6395	0.6728	0.9034	0.3419
Baseline cHAMD-17	0.0843	0.0296	8.1192	0.0044	-0.0073	0.0305	0.0579	0.8098
Baseline-to-week-4 change in cHAMD-17	3.5097	0.6256	31.4782	<0.001	0.0575	0.0399	31.2506	<0.001
Baseline CAST-IRR	-0.0608	0.0375	2.6321	0.1047	-3.5835	0.6410	2.0696	0.1503
Baseline-to-week-4 change in CAST-IRR	2.0955	0.5960	12.3623	0.0004	-1.3869	0.6351	4.7684	0.0290

Footnote: We computed 17-item Hamilton Depression Rating scale (cHAMD-17) using the following formula: HAMD-17 = 0.11 + 0.53*(IDS-C), per Vittengl et al. (15) in the Combining Medications to Enhance Depression Outcomes (CO-MED) trial. Remission and no-meaningful-benefit at week-8 were defined as cHAMD-17 ≤7 and <30% reduction in cHAMD-17 from baseline respectively. Irritability was measured with the irritability domain of Concise Associated Symptom Tracking scale (CAST-IRR).
